# Supplementary material for: The voluntary control of piloerection
Source: PeerJ. 2018 Jul 30;6:e5292. doi: 10.7717/peerj.5292 (PMC6071615; doi:10.7717/peerj.5292)
Supplement: Supplemental Information 2 [file peerj-06-5292-s003.pdf]

Supplementary materials – study questionnaire

Q1 How old are you?

Q2 How old were you when you discovered you could give yourself goosebumps? (Approximate is OK)

Q3 (Optional) Please briefly describe the situation you were in when you first discovered that you could give yourself goosebumps?

Q4 What is your biological sex?
❍ Male (1)
❍ Female (2)

Q5 Choose from the following list the sites where the sensation of goosebumps typically begins(more than one may apply)
❑ Top or sides of head inc. ears (1)
❑ Back of head/neck (2)
❑ Shoulders (3)
❑ Upper back (4)
❑ Lower back (5)
❑ Chest/abdomen (6)
❑ Crotch (7)
❑ Arms (8)
❑ Legs (9)
❑ Hands (10)
❑ Feet (11)
❑ Other (12) ____________________

Q6 Check off ALL the sites where you typically experience goosebumps(more than one may apply)
❑ Top or sides of head inc. ears (1)
❑ Back of head/neck (2)
❑ Shoulders (3)
❑ Upper back (4)
❑ Lower back (5)
❑ Chest/abdomen (6)
❑ Crotch (7)
❑ Arms (8)
❑ Legs (9)
❑ Hands (10)
❑ Feet (11)
❑ Other: (12) ____________________

Q7 Where is the sensation strongest or most noticeable?(more than one may apply)
- ❑ Top or sides of head inc. ears (1)
- ❑ Back of head/neck (2)
- ❑ Shoulders (3)
- ❑ Upper back (4)
- ❑ Lower back (5)
- ❑ Chest/abdomen (6)
- ❑ Crotch (7)
- ❑ Arms (8)
- ❑ Legs (9)
- ❑ Hands (10)
- ❑ Feet (11)
- ❑ Other: (12) ____________________

Q8 How long does it take the 'goosebump' response to start after you initiate it?
- ❍ 0 to 1 second (1)
- ❍ 2 to 4 seconds (2)
- ❍ 5 to 10 seconds (3)
- ❍ 10+ seconds (4)

Q9 Say you turned your goosebumps all the way on and then all the way off again. How long would that take?
- ❍ 0 to 3 second (1)
- ❍ 3 to 6 seconds (2)
- ❍ 6 to 9 seconds (3)
- ❍ 9 to 12 seconds (4)
- ❍ 12 to 15 seconds (5)
- ❍ Longer than 15 seconds (6)

Q10 Can you control the location of the goosebumps?
- ❍ Yes (1)
- ❍ No (2)

Q11 Do your goosebumps demonstrate 'sidedness'? As in, are they ever stronger, more noticeable or different between your left and right hand sides?
- ❍ Yes (1)
- ❍ No (2)

Q12 Do you find it harder to create goosebumps on one side than the other?
- ❍ Yes (1)
- ❍ No (2)

Q13 When you found out that other people typically cannot produce goosebumps at will, were you surprised?This could be recent or in the past.

- ❍ Very surprised (1)
- ❍ Somewhat surprised (2)
- ❍ A little surprised (3)
- ❍ Not surprised at all (4)

Q14 How hard do you find the act of producing goosebumps?

- ❍ Very easy or effortless (1)
- ❍ Requires some concentration or focus (2)
- ❍ Requires a lot of concentration or focus (3)

Q15 How do you produce the goosebumps?

- ❍ I do something, it's a physical thing - like moving my hands, or blinking. I don't need to think of anything. (1)
- ❍ I think of something good, or sad, or scary - it's a mental thing. I don't need to do or move anything. (2)
- ❍ I can do it either way. (3)
- ❍ Other: (4) ____________________

Q16 Can you produce goosebumps under the following situations? (select any that apply)You can try them out now if you don't know...

- ❑ While you're breathing in? (1)
- ❑ While you're breathing out? (2)
- ❑ While you're holding your breath? (3)
- ❑ While your lungs are empty? (4)

Q17 Which of the following is easiest?

- ❍ Producing goosebumps breathing in (1)
- ❍ Producing goosebumps breathing out (2)
- ❍ Producing goosebumps while holding breath (3)
- ❍ While your lungs are empty (4)
- ❍ There is no difference between any of them (5)

Q18 How commonly have you practiced the ability to give yourself goosebumps?

- ❍ Frequently (at least once a week) (1)
- ❍ Occasionally (at least once a month) (2)
- ❍ Rarely (less than once a month) (3)
- ❍ I Never or almost never practice (4)

Q19 We are interested in the feelings and thoughts that accompany voluntarily induced goosebumps. Please choose any of the feelings and thoughts that accompany voluntarily induced goosebumps. (more than one may apply)

- ❑ feel a sense of awe and wonder (1)
- ❑ feel touched (2)
- ❑ feel like crying (3)
- ❑ feel absorbed and immersed (4)
- ❑ completely lose track of time (5)
- ❑ feel like you are somewhere else (6)
- ❑ feel detached from your surroundings (7)

Q20 Do you ever use your ability to produce goosebumps in the following situations: (more than one may apply)

- ❑ listening to music (1)
- ❑ watching films or TV series (2)
- ❑ viewing art (3)
- ❑ reading literature, or poetry, or quotations (4)
- ❑ during creative activities (e.g. painting) (5)
- ❑ watching dance (6)
- ❑ theater (7)
- ❑ playing computer games (8)
- ❑ other (please list any other activities) (9) ____________________

Q21 Please think of an area of the arts (e.g. music, visual art, reading, watching dance etc) you encounter most often in your daily life. What area of the arts is that for you?

Q22 With reference to this area of the arts, how often do you . . .

	Never or rarely (1)	2 (2)	3 (3)	4 (4)	5 (5)	6 (6)	Almost always (7)
feel absorbed and immersed (1)	❍	❍	❍	❍	❍	❍	❍
completely lose track of time (2)	❍	❍	❍	❍	❍	❍	❍
feel chills down your spine (3)	❍	❍	❍	❍	❍	❍	❍
get goose bumps (4)	❍	❍	❍	❍	❍	❍	❍
feel like you're somewhere else (5)	❍	❍	❍	❍	❍	❍	❍
feel like your hair is standing on end (6)	❍	❍	❍	❍	❍	❍	❍
feel like crying (7)	❍	❍	❍	❍	❍	❍	❍
feel touched (8)	❍	❍	❍	❍	❍	❍	❍
feel detached from your surroundings (9)	❍	❍	❍	❍	❍	❍	❍
feel a sense of awe and wonder (10)	❍	❍	❍	❍	❍	❍	❍

Q23 What stimuli or situations usually elicit involuntary goosebumps? (free response)

Q24 Do you tend to make these experiences last longer by prolonging the goosebumps yourself?

❍ Yes (1)

❍ No (2)

Q25 What's YOUR own explanation for being able to give yourself goosebumps?

Q26 In your own words, describe the whole process of how you give yourself goosebumps.

Q27 Here are a number of personality traits that may or may not apply to you. Please select a response next to each statement to indicate the extent to which you agree or disagree with that statement. You should rate the extent to which the pair of traits applies to you, even if one characteristic applies more strongly than the other. I see myself as...

	Disagree strongly (1)	Disagree moderately (2)	Disagree a little (3)	Neither agree nor disagree (4)	Agree a little (5)	Agree moderately (6)	Agree strongly (7)
Extraverted, enthusiastic (1)	❍	❍	❍	❍	❍	❍	❍
Critical, quarrelsome (2)	❍	❍	❍	❍	❍	❍	❍
Dependable, self-disciplined (3)	❍	❍	❍	❍	❍	❍	❍
Anxious, easily upset (4)	❍	❍	❍	❍	❍	❍	❍
Open to new experiences, complex (5)	❍	❍	❍	❍	❍	❍	❍
Reserved, quiet (6)	❍	❍	❍	❍	❍	❍	❍
Sympathetic, warm (7)	❍	❍	❍	❍	❍	❍	❍
Disorganized, careless (8)	❍	❍	❍	❍	❍	❍	❍
Calm, emotionally stable (9)	❍	❍	❍	❍	❍	❍	❍
Conventional, uncreative (10)	❍	❍	❍	❍	❍	❍	❍

Q25 Here are a number of characteristics that may or may not describe you. Please click the number that best indicates the extent to which you agree or disagree with each statement listed below. Be as honest as possible, but rely on your initial feeling and do not think too much about each item.

	Strongly agree (1)	Agree (2)	Neither Agree nor Disagree (3)	Disagree (4)	Strongly Disagree (5)
I am quick to understand things. (1)	❍	❍	❍	❍	❍
I believe in the importance of art. (2)	❍	❍	❍	❍	❍
I have difficulty understanding abstract ideas. (3)	❍	❍	❍	❍	❍
I enjoy the beauty of nature. (4)	❍	❍	❍	❍	❍
I can handle a lot of information. (5)	❍	❍	❍	❍	❍
I love to reflect on things. (6)	❍	❍	❍	❍	❍
I like to solve complex problems. (7)	❍	❍	❍	❍	❍
I get deeply immersed in music. (8)	❍	❍	❍	❍	❍
I avoid philosophical discussions. (9)	❍	❍	❍	❍	❍
I do not like poetry. (10)	❍	❍	❍	❍	❍
I avoid difficult reading material. (11)	❍	❍	❍	❍	❍
I seldom notice the emotional aspects of paintings and pictures. (12)	❍	❍	❍	❍	❍
I have a rich vocabulary. (13)	❍	❍	❍	❍	❍
I need a creative outlet. (14)	❍	❍	❍	❍	❍
I think quickly. (15)	❍	❍	❍	❍	❍
I seldom get lost in thought. (16)	❍	❍	❍	❍	❍
I learn things slowly. (17)	❍	❍	❍	❍	❍
I seldom daydream. (18)	❍	❍	❍	❍	❍
I formulate ideas clearly. (19)	❍	❍	❍	❍	❍
I see beauty in things that others might not notice. (20)	❍	❍	❍	❍	❍
